# Impact of serum 1,5-anhydro-d-glucitol level on the prediction of severe coronary artery calcification: an intravascular ultrasound study

**DOI:** 10.1186/s12933-019-0878-1

**Published:** 2019-06-03

**Authors:** Hideki Wada, Tomotaka Dohi, Katsumi Miyauchi, Norihito Takahashi, Hirohisa Endo, Yoshiteru Kato, Manabu Ogita, Iwao Okai, Hiroshi Iwata, Shinya Okazaki, Kikuo Isoda, Kazunori Shimada, Satoru Suwa, Hiroyuki Daida

**Affiliations:** 1 Department of Cardiovascular Medicine, Juntendo University Shizuoka Hospital, Izunokuni, Shizuoka Japan; 2 Department of Cardiovascular Medicine, Juntendo University Graduate School of Medicine, 2-1-1 Hongo, Bunkyo-ku, Tokyo, 113-0033 Japan

**Keywords:** 1,5-Anhydro-d-glucitol, Postprandial hyperglycemia, Coronary artery disease, Coronary calcification, Intravascular ultrasound imaging

## Abstract

**Background:**

A low 1,5-anhydro-d-glucitol (AG) blood level is considered a clinical marker of postprandial hyperglycemia. Previous studies reported that 1,5-AG levels were associated with vascular endothelial dysfunction and coronary artery disease (CAD). However, the association between 1,5-AG levels and coronary artery plaque in patients with CAD is unclear.

**Methods:**

This study included 161 patients who underwent percutaneous coronary intervention for CAD. The culprit plaque characteristics and the extent of coronary calcification, which was measured by the angle of its arc, were assessed by preintervention intravascular ultrasound (IVUS). Patients with chronic kidney disease or glycosylated hemoglobin ≥ 7.0 were excluded. Patients were divided into 2 groups according to serum 1,5-AG levels (< 14.0 μg/mL vs. ≥ 14 μg/mL).

**Results:**

The total atheroma volume and the presence of IVUS-attenuated plaque in the culprit lesions were similar between groups. Calcified plaques were frequently observed in the low 1,5-AG group (p = 0.06). Compared with the high 1,5-AG group, the low 1,5-AG group had significantly higher median maximum calcification (144° vs. 107°, p = 0.03) and more frequent calcified plaques with a maximum calcification angle of ≥ 180° (34.0% vs. 13.2%, p = 0.003). Multivariate logistic regression analysis showed that a low 1,5-AG level was a significant predictor of a greater calcification angle (> 180°) (OR 2.64, 95% CI 1.10–6.29, p = 0.03).

**Conclusions:**

Low 1,5-AG level, which indicated postprandial hyperglycemia, was associated with the severity of coronary artery calcification. Further studies are needed to clarify the effects of postprandial hyperglycemia on coronary artery calcification.

## Background

Type 2 diabetes mellitus (DM) is a complex disease that is related to atherosclerotic diseases. Patients with type 2 DM have a higher incidence of cardiovascular disease (CVD) compared with the general population [1, 2]. Increased glycosylated hemoglobin (HbA1c), which is a common marker of long-term glycemic control, had been associated with poor prognosis in patients with DM [3]. Moreover, previous studies demonstrated that postprandial hyperglycemia affected mortality and CVD progression [4–6]. 1,5-anhydro-d-glucitol (1,5-AG) is a naturally occurring 1-deoxy form of glucose and thought to originate in the diet [7]. Serum 1,5-AG levels have been known to rapidly decrease along with the excretion of glucose in the urine and were reported to better reflect short-term glucose control and postprandial hyperglycemia, compared with HbA1c levels [8, 9]. Some studies have shown that 1,5-AG levels were associated with vascular endothelial dysfunction [10] and superior to HbA1c in detecting the presence of CVD [11, 12]. Our colleagues demonstrated that low 1,5-AG levels predicted the long-term clinical outcomes in CVD patients with HbA1c levels < 7.0% [13, 14]. Therefore, 1,5-AG level might be a useful predictive marker in patients with relatively low HbA1c levels. However, the mechanisms of the relationship between CVD and 1,5-AG level are unclear. In this study, we sought to evaluate the association between 1,5-AG level and culprit coronary plaque findings, including quantitative and qualitative analyses by gray-scale intravascular ultrasound (IVUS), in patients with CVD who underwent percutaneous coronary intervention (PCI).

## Methods

### Study population

**Fig. 1:**
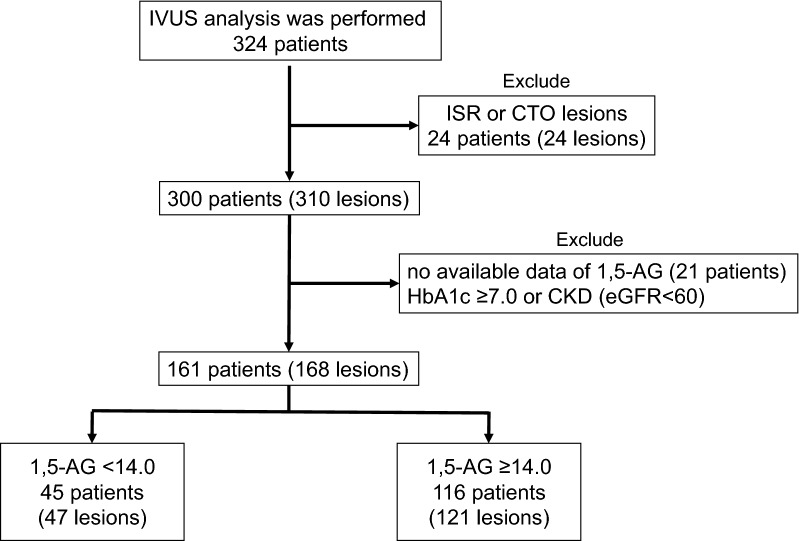
Flow chart of the study inclusion IVUS analysis was performed in 324 patients between August 2014 and August 2016. A total of 161 patients (168 lesions) were enrolled. *1,5-AG* 1,5-anhydro-d-glucitol, *CKD* chronic kidney disease, *CTO* chronic total occlusion, *eGFR* estimated glomerular filtration rate, *ISR* in-stent restenosis, *IVUS* intravascular ultrasound, *PCI* percutaneous coronary intervention

In this prospective, single-center, observational study, 324 consecutive coronary artery disease (CAD) patients who were admitted to Juntendo University Hospital were enrolled from August 2014 to August 2016 (Fig. 1). The inclusion criteria were (1) patients who underwent PCI under IVUS guidance to treat a de novo culprit lesion and (2) patients who were evaluated using IVUS. The exclusion criteria were (1) patients who had in-stent restenosis or a lesion with chronic total occlusion; (2) patients who had chronic kidney disease (CKD), which was defined as an estimated glomerular filtration rate of < 60 mL/min/1.73 m^2^ that was calculated using the Modification of Diet in Renal Disease equation, modified with a Japanese coefficient using baseline serum creatinine [15]; (3) patients who had HbA1c levels ≥ 7.0% and those under treatment with sodium-glucose co-transporter 2 inhibitors; and (4) patients in whom adequate IVUS images or data on 1,5-AG levels were not obtained. Patients were divided into 2 groups (low 1,5-AG group, < 14.0 µg/mL; high 1,5-AG group, ≥ 14.0 µg/mL).

This study was approved by the Juntendo University ethics committee and was performed in accordance with the Declaration of Helsinki. All participants provided written, informed consent.

### Data collection and blood sampling

Data on demographics, CAD risk factors, and medication use were collected from our institutional database. Blood samples were collected in the early morning after overnight fasting, and blood pressure (BP) was measured on admission. Patients with BP > 140/90 mmHg or those receiving antihypertensive drugs were regarded as hypertensive. Dyslipidemia was defined as low-density lipoprotein cholesterol ≥ 140 mg/dL, high-density lipoprotein cholesterol ≤ 40 mg/dL, triglycerides ≥ 150 mg/dL, or current treatment with statins and/or lipid-lowering agents [16]. DM was defined as either an HbA1c ≥ 6.5% or use of medications, such as insulin or oral hypoglycemic drugs. A current smoker was defined as a person who was a smoker at the time of PCI or who had quit smoking within 1 year before PCI. Acute coronary syndrome (ACS) was defined as acute myocardial infarction (MI) and unstable angina. Acute MI was characterized by elevated cardiac enzymes. Unstable angina was diagnosed in the presence of ischemic symptoms, without the elevation of the enzymes and biomarkers associated with myocardial necrosis.

To measure 1,5-AG levels, blood samples were obtained immediately prior to coronary angiography and stored at − 80 °C until measurement. Serum 1,5-AG levels were measured with a colorimetric method (Nippon Kayaku, Tokyo, Japan) using a Lana 1,5-AG auto liquid automatic analyzer (JCA-BM 8060, JEOL Ltd., Tokyo, Japan).

### IVUS image acquisition and analysis

In all study patients, the culprit plaque lesion was evaluated by preintervention IVUS. By comparing pre- and post-PCI IVUS findings, a culprit lesion segment was defined as the lesion that was stented. In this study, the mechanical rotating 40-MHz transducer that was used were from 1 commercially available IVUS systems and catheters (Atlantis Pro2, Boston Scientific Corporation, Natick, MA, USA; View It, Terumo Corporation, Tokyo, Japan). After intracoronary administration of 0.1–0.2 mg nitroglycerin, IVUS imaging of the culprit lesion segment was performed before balloon dilatation or after small (1.5–2.0 mm) balloon dilatation. All IVUS pullback maneuvers were performed automatically at 0.5 mm/s. All measurements were fulfilled at the end of this study. Quantitative and qualitative IVUS analyses were performed according to the criteria of the American College of Cardiology Clinical Expert Consensus Document on Standards for Acquisition, Measurement and Reporting of Intravascular Ultrasound Studies [17]. The morphological features were diagnosed by careful review of the IVUS images and upon the agreement of the 2 independent experienced cardiologists (H.W. and T.D.) who were blinded to the clinical data. Offline analyses of all imaged segments were performed using computerized planimetry software (QIVUS; Medis Medical Imaging System, Leiden, the Netherlands).

**Fig. 2:**
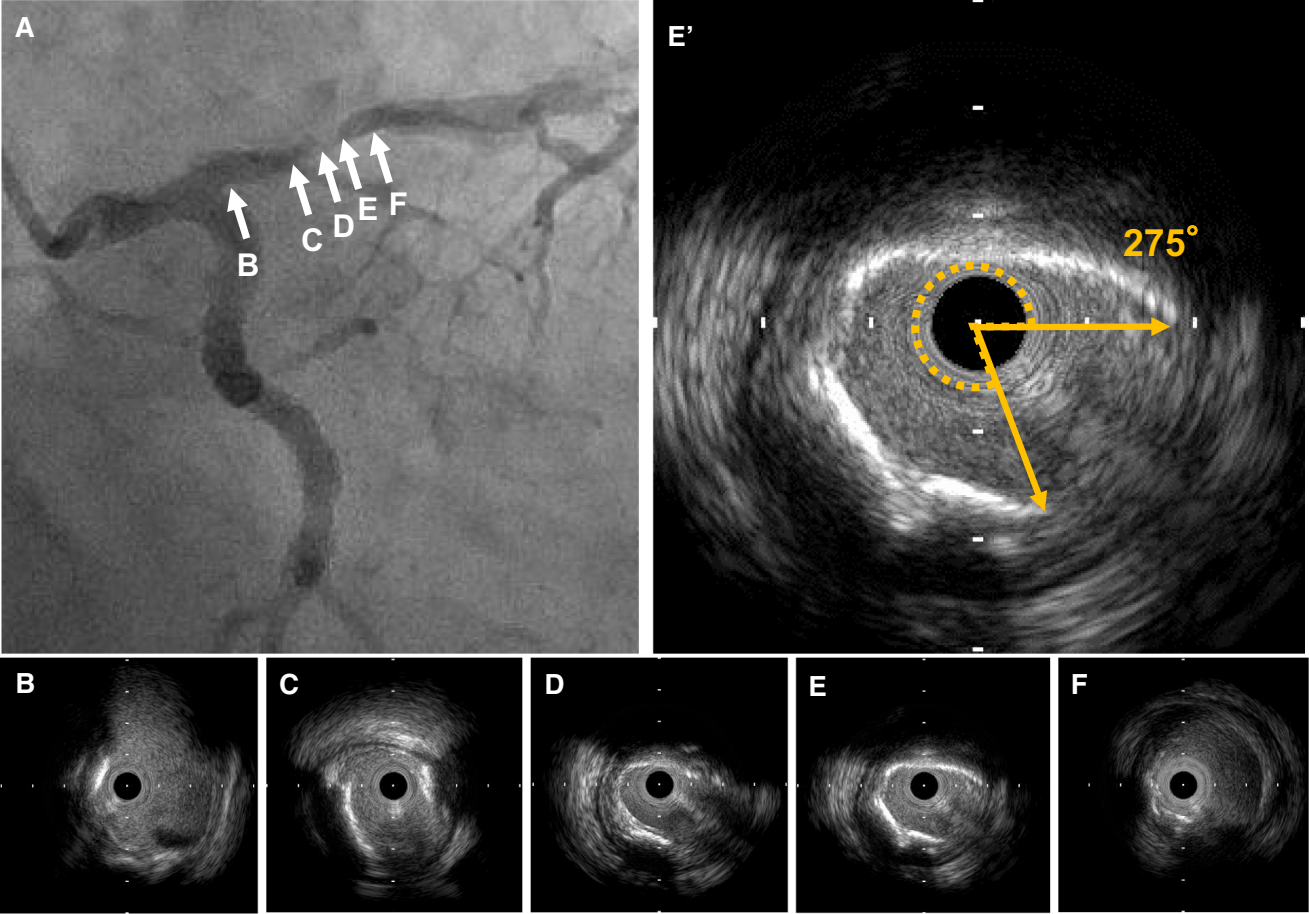
Representative case of intravascular ultrasound-detected attenuated plaque. A 70-year-old man with stable coronary artery disease is shown to have severe coronary artery stenosis on angiography (**A**). Intravascular ultrasound examination **B**–**F** shows a severely calcified plaque (maximum calcification angle, 275°) in the proximal left descending artery (**E**). In this case, the serum 1,5-anhydro-d-glucitol level is 8.3 µg/mL

The quantitative IVUS measurements included the external elastic membrane (EEM), lumen cross-sectional area (CSA), and the plaque plus media CSA (EEM–lumen CSA). The plaque burden was calculated as the plaque plus media CSA divided by the lesion EEM CSA multiplied by 100. Total atheroma volume (TAV) was calculated as the sum of the differences between the EEM and the luminal areas across all segments analyzed. Percent atheroma volume was calculated as the proportion of the entire lesion segment occupied by the atherosclerotic plaque. The qualitative IVUS variables included plaque rupture (presence of a cavity that communicated with the lumen with an overlying residual fibrous cap); thrombus (an intraluminal mass, often with a layered, lobulated, or pedunculated appearance); calcification (brighter plaque than adventitia with acoustic shadowing); and ultrasound attenuation behind the plaque in the absence of calcification. The maximum angle of the ultrasound attenuation and the calcification of the lesion were also measured. The remodeling index was calculated as the EEM CSA at the minimal lumen area site divided by the average of the proximal and distal reference EEM CSA. A representative case is shown in Fig. 2.

### Statistical analysis

Quantitative data are presented as mean ± standard deviation (SD) or median [interquartile range (IQR)]. Categorical variables are presented as frequencies. Continuous variables were compared using unpaired t-test or the Mann–Whitney U-test, as appropriate. Categorical variables were compared using the Chi-square test. A logistic regression analysis was used to compare the odds ratios (ORs) of the calcified plaque (maximum calcified angle ≥ 180°) between 2 groups (low 1,5-AG and high 1,5-AG). Differences were considered significant at p < 0.05. Statistical analyses were carried out using JMP version 12.0 software (SAS Institute, Cary, NC, USA).

## Results

### Baseline patient clinical characteristics

In the 161 patients, the median and mean 1,5-AG were 17.6 (IQR 13.4, 23.3) and 18.6 ± 7.1, respectively; of these, 45 patients (28.0%) were assigned to the low 1,5-AG group. The baseline demographics are summarized in Table 1. The mean age of the patients was 64.9 years, 86.3% were men, and the rate of DM was 19.9%. Compared with the patients in the high 1,5-AG group, those in the low 1,5-AG group were more likely to have DM and have higher fasting blood glucose levels. On the other hand, there was no significant difference in the HbA1c levels between the low 1,5-AG and high 1,5-AG groups.

**Table 1:** Mean baseline characteristics of the patients

	Overall n = 161	Low 1,5-AG group < 14.0 μg/mL n = 45	High 1,5-AG group ≥ 14.0 µg/mL n = 116	p value
1,5-Anhydro-d-glucitol, μg/mL^a^	17.6 [13.4, 23.3]	11.0 [8.6, 12.5]	20.6 [17.3, 25.5]	< 0.0001
Age, years	64.9 ± 11.2	66.3 ± 10.6	64.4 ± 11.4	0.35
Male (%)	139 (86.3)	38 (84.4)	101 (87.1)	0.66
Body mass index, kg/m^2^	24.8 ± 3.7	24.8 ± 3.7	24.8 ± 3.8	0.96
Hypertension (%)	111 (68.9)	31 (68.9)	80 (69.0)	0.99
Diabetes (%)	32 (19.9)	16 (35.6)	16 (13.8)	0.002
Dyslipidemia (%)	114 (70.8)	32 (71.1)	82 (70.7)	0.96
Current smoker (%)	41 (25.5)	11 (24.4)	30 (25.9)	0.85
Family history of coronary artery disease (%)	45 (28.1)	14 (31.1)	31 (27.0)	0.60
Acute coronary syndrome presentation	30 (18.6)	9 (20.0)	21 (18.1)	0.78
Oral hypoglycemic drugs	20 (12.4)	11 (24.4)	9 (7.8)	0.006
Metformin (%)	1 (0.6)	1 (2.2)	0 (0.0)	0.11
Sulfonylureas (%)	5 (3.1)	5 (11.1)	0 (0.0)	0.0003
Dipeptidyl peptidase-4 inhibitors (%)	17 (10.6)	11 (24.4)	6 (5.2)	0.0008
Others (%)	5 (3.1)	2 (4.4)	3 (2.6)	0.56
Prior statin use, %	120 (74.5)	30 (66.7)	90 (77.6)	0.15
Low-density lipoprotein cholesterol, mg/dL	89.0 ± 23.9	91.4 ± 23.1	88.0 ± 24.3	0.46
High-density lipoprotein cholesterol, mg/dL	45.3 ± 12.1	44.6 ± 11.8	45.6 ± 12.3	0.64
Triglycerides, mg/dL	126.6 ± 66.8	119.5 ± 67.0	129.4 ± 66.8	0.40
Hemoglobin A1c, %	5.9 ± 0.4	6.0 ± 0.5	5.8 ± 0.4	0.10
Fasting blood glucose, mg/dL	97.3 ± 19.0	106.6 ± 25.8	93.7 ± 14.2	< 0.0001
High-sensitivity C-reactive protein, mg/dL^a^	0.05 [0.02, 0.13]	0.05 [0.02, 0.12]	0.05 [0.03, 0.15]	0.54
Estimated glomerular filtration rate, mL/min/1.73 m^2^	83.5 ± 17.9	85.1 ± 19.8	82.9 ± 17.2	0.48

^a^Median [interquartile range]

### Quantitative and qualitative IVUS findings

A total of 168 culprit lesions were analyzed. The lesion-level findings on coronary angiography and IVUS are shown in Table 2. The overall median minimal lumen area, percentage atheroma volume, and remodeling index were 2.1 mm^2^, 60.8%, and 1.1, respectively. There were no significant differences in the quantitative parameters, such as plaque burden at the minimal lumen area, percent atheroma volume, total atheroma volume, and remodeling index between the low 1,5-AG and high 1,5-AG groups.

**Table 2:** Mean coronary angiography and IVUS findings of the lesions

	Overall (n = 168)	Low 1,5-AG group < 14.0 μg/mL (n = 47)	High 1,5-AG group ≥ 14.0 μg/mL (n = 121)	p value
Quantitative coronary angiography				
Culprit vessel				0.83
Left anterior descending artery (%)	84 (50.0)	24 (51.1)	60 (49.6)	
Right coronary artery (%)	48 (28.6)	15 (31.9)	33 (27.3)	
Left circumflex artery (%)	31 (18.5)	7 (14.9)	24 (19.8)	
Others	5 (3.0)	1 (2.1)	4 (3.3)	
Total length of the lesions, mm^a^	23.9 [16.1, 32.1]	24.1 [15.9, 36.8]	23.6 [17.7, 32.0]	0.90
IVUS findings				
Quantitative parameters				
MLA, mm^2 a^	2.1 [1.8, 2.7]	2.1 [1.8, 2.6]	2.1 [1.7, 2.7]	0.45
EEM at MLA site, mm^2 a^	12.3 [9.5, 16.3]	12.0 [8.4, 17.8]	12.4 [9.5, 15.9]	0.88
Area of stenosis at the MLA, %^a^	81.8 [75.5, 87.3]	78.6 [74.3, 88.5]	81.9 [76.0, 87.2]	0.41
Percent atheroma volume, %^a^	60.8 [53.9, 67.5]	61.9 [54.3, 67.1]	60.1 [53.6, 68.4]	0.81
Total atheroma volume, mm^3 a^	194.8 [139.9, 287.0]	206.9 [140.3, 320.9]	188.7 [136.1, 264.1]	0.52
Total atheroma volume_normalized_, mm^3 a^	205.9 [146.6, 264.2]	216.4 [137.4, 278.3]	205.2 [147.2, 253.9]	0.57
Remodeling index^a^	1.1 [1.0, 1.2]	1.1 [1.0, 1.2]	1.1 [1.0, 1.2]	0.83
Qualitative assessment and parameters				
Culprit plaque type				0.31
Soft	63 (37.5)	19 (40.4)	44 (36.4)	
Fibrous	45 (26.8)	8 (17.0))	37 (30.6)	
Calcified	29 (17.3)	10 (21.3)	19 (15.7)	
Mixed	31 (18.5)	10 (21.3)	21 (17.4)	
Plaque rupture (%)	39 (23.2)	14 (29.8)	25 (20.7)	0.21
Thrombosis on IVUS (%)	18 (10.7)	6 (12.8)	12 (9.9)	0.60
Calcified nodule (%)	21 (12.5)	6 (12.8)	15 (12.4)	0.95
Ultrasound attenuation (%)	103 (61.3)	31 (66.0)	72 (59.5)	0.44
Maximum attenuation angle, ° ^a^	137 [108, 178]	141 [108, 187]	136 [110, 172]	0.99
Calcified lesion (%)	103 (61.3)	34 (72.3)	69 (57.0)	0.06
Maximum calcium angle, ° ^a^	115 [84, 228]	144 [88, 276]	107 [80, 156]	0.03
Maximum calcium arch ≥ 130° (%)	45 (26.8)	19 (40.4)	26 (21.5)	0.01
Maximum calcium arch ≥ 180° (%)	32 (19.1)	16 (34.0)	16 (13.2)	0.002

*EEM* external elastic membrane, *IVUS* intravascular ultrasound, *MLA* minimal lumen area

^a^Median [interquartile range]

In the overall qualitative analysis, 37.5% and 17.3% of the plaques were described as soft and calcific, respectively. There was no significant difference in the culprit plaque type between the groups. Similarly, the frequency of plaque rupture and calcified nodules did not differ between the groups. The frequency of calcified lesion tended to be higher in the low 1,5-AG group than in the high 1,5-AG group (72.3% vs. 57.0%; p = 0.06); whereas the frequency of ultrasound attenuation was similar between the groups. The median maximum calcification angle was significantly higher in the low 1,5-AG group than in the high 1,5-AG group (144° vs. 107°, p = 0.03). Plaques with a maximum calcification angle of > 180° were more frequently observed in the low 1,5-AG group than in the high 1,5-AG group (34.0% vs. 13.2%; p = 0.01).

### Multivariate logistic regression analysis

The univariate logistic regression analysis showed that low 1,5-AG level was significantly associated with a greater calcification angle (> 180°) (OR 3.39, 95% CI 1.52–7.60; p = 0.003). To determine the value of serum 1,5-AG, a multivariate logistic regression analysis was conducted. The variables for which the unadjusted p value was < 0.10 in the univariate analysis were included in the multivariate model for greater calcification angle. In the multivariate model (Table 3), the independent predictors of a greater calcification angle were low 1,5-AG level (adjusted OR 2.64, 95% confidence interval (CI) 1.10–6.29; p = 0.03) and the presence of diabetes (OR 2.99, 95% CI 1.20–7.33; p = 0.02).

**Table 3:** Multivariate logistic regression analysis for the factors that affect maximum calcification angle ≥ 180°

	Univariate			Multivariate		
	Odds ratio	95% CI	p value	Odds ratio	95% CI	p value
1,5-Anhydro-d-glucitol < 14.0 μg/mL	3.39	1.52–7.60	0.003	2.64	1.10–6.29	0.03
Acute coronary syndrome on admission	0.42	0.10–1.29	0.14			
Age	1.04	1.01–1.08	0.03	1.04	0.996–1.081	0.08
Body mass index	0.97	0.87-1.08	0.59			
Current smoker	1.00	0.39–2.36	1.00			
Diabetes	4.03	1.74–9.33	0.001	2.99	1.20–7.33	0.02
Dyslipidemia	1.54	0.64–4.12	0.34			
Family history	0.90	0.37–2.05	0.80			
Hypertension	2.76	1.07–8.56	0.03	2.60	0.95–8.45	0.06
Male	0.82	0.30–2.67	0.73			
Statin use on admission	0.68	0.30–1.63	0.37			

*CI* confidence interval

## Discussion

In the present study, the association between 1,5-AG level and culprit coronary plaque findings in CVD patients who underwent PCI was examined. The major findings of the present study were (1) compared with patients in the high 1,5-AG group, those in the low 1,5-AG group were more likely to have a higher fasting blood glucose levels despite similar HbA1c levels; (2) plaques with a maximum calcification angle of > 180° were more frequently observed in the low 1,5-AG group than in the high 1,5-AG group; and (3) low 1,5-AG level was an independent predictor of a greater calcification angle. Overall, our results showed that low 1,5-AG was associated with more severe plaque calcification, which is a discriminator of plaque atherosclerosis.

1,5-AG is a monosaccharide that can be primarily obtained from dietary sources. The concentrations of 1,5-AG in blood and tissues are maintained constant, because the amount filtered by the glomeruli is reabsorbed in the renal proximal tubule. Reabsorption is competitively inhibited by glucose. In the setting hyperglycemia, 1,5-AG is excreted in the urine, and its serum level decreases rapidly [7]. Therefore, serum 1,5-AG level is considered a clinical marker of short-term glycemic status. In addition, 1,5-AG had been reported to reflect glycemic excursions, often in the postprandial state, more robustly than HbA1c, in patients with moderately controlled DM [8]. No differences in 1,5-AG levels were found in patients with markedly different estimated glomerular filtration rates [18], and these levels were not influenced by administration of a gliptin compound [19]. Serum 1,5-AG concentration is a marker for hyperglycemia and may be particularly useful as an indicator for short-term glycemic excursions [20] and is significantly higher in DM patients [21]. Finally, 1,5-AG might suppress the blood glucose elevation through inhibition of sucrase and intestinal glucose absorption [22].

HbA1c is a well-known useful maker for the diagnosis of DM and glycemic control. Elevated HbA1c had been associated with increased risk of cardiovascular events [3, 23, 24], and a target of < 7.0% had been established by the American Diabetes Association [25]. However, despite the beneficial effects of lowering HbA1c level to < 7.0% on macrovascular disease, the risks for macrovascular complications remain [26, 27]. Accordingly, comprehensive evaluation, in addition to that of HbA1c, may be necessary for the prevention of macrovascular diseases, especially in patients with relatively low HbA1c levels.

Our colleagues have demonstrated that low 1,5-AG levels predicted the long-term clinical outcomes in both ACS and stable CAD patients with HbA1c levels < 7.0% [13, 14]. They considered that 1,5-AG, which detected postprandial hyperglycemia, may be a useful marker for risk stratification in CAD patients with HbA1c < 7.0%. In the present study, we showed that a lower 1,5-AG was associated with more severe plaque calcification, as detected by IVUS imaging. Interestingly, coronary atheroma volume or plaque burden were not significantly associated with serum 1,5-AG levels. Postprandial hyperglycemia might not affect plaque quantitative parameters among patients with relatively low HbA1c levels and preserved renal function. Coronary calcification had been recognized to be associated with atherosclerosis [28] and is an established predictor of future cardiac events [29–31]. Coronary artery calcification is generally related with the extent of CAD. In a histologic study, Sangiorgi et al. reported a significant association between coronary calcification area and plaque volume [32]. In addition, studies on IVUS and computed tomography (CT) showed an association between coronary artery calcification and extent of atherosclerosis plaque [33, 34]. Coronary artery calcification tended to be higher in patients with DM, correlated with total plaque burden, and was an independent risk factor for adverse cardiac events [34, 35]. Medial artery calcification, which is a non-obstructive condition leading to related artery compliance, is known a risk factor of future cardiovascular events among patients with DM [36]. Previous in vivo experiments reported that diabetes promote vascular calcification in part by recruiting adventitial cell with osteogenic potential [37]. So, osteopontin, an osteoblast matrix protein, is thought to play a role in the development of vascular complication among patients with diabetes [38]. Previous studies have shown the association between elevated HbA1c and coronary artery calcification using CT [39, 40]. In a study on 2076 participants without DM, Carson et al. evaluated the predictive value of HbA1c using CT at baseline and after a 5-year follow-up [39]. They showed that higher HbA1c was independently associated with advanced coronary artery calcification and calcification progression. Our study demonstrated that low 1,5-AG level was related with the incidence of severe coronary calcification, independent of the other risk factors. Furthermore, patients with CKD, which had been recognized a strong risk factor for coronary artery calcification, were excluded in the present study. Therefore, among patients with relatively low HbA1c and without CKD, 1,5-AG might be useful marker to predict the incidence of severe coronary artery calcification and future cardiovascular events.

This study had several limitations. First, as a single-center, observational study on a small patient cohort, unknown confounding factors might have affected the outcomes. Studies with large sample size and racial diversity would be more effective in evaluating the value of 1,5-AG levels. Second, we detected lesion calcifications using gray-scale IVUS only. Although the calcium thickness or volume could not be calculated by IVUS, these can be easily used to detect lesion calcification [41, 42]. Finally, we don’t have any vitro experiments that would help support the results of the present study. Previous study demonstrated that advanced glycation end products induce calcification of vascular smooth muscle cells by osteoblast-like differentiation of smooth muscle cells [43]. Further studies to prove the association between low 1,5-AG levels and coronary calcification are needed.

## Conclusions

This study found that low 1,5-AG level, which indicated postprandial hyperglycemia, was associated with the severity of coronary artery calcification. Further studies are needed to confirm the independent effects of postprandial hyperglycemia on coronary artery calcification.

## Data Availability

The datasets during and/or analyzed during the current study are available from the corresponding author with reasonable request.

## Abbreviations

ACS: acute coronary syndrome
1,5-AG: 1,5-anhydro-d-glucitol
BP: blood pressure
CAD: coronary artery disease
CI: confidence interval
CSA: cross-sectional area
CT: computed tomography
CVD: cardiovascular disease
DM: diabetes mellitus
EEM: external elastic membrane
IQR: interquartile range
HbA1c: glycosylated hemoglobin
IVUS: intravascular ultrasound
MI: myocardial infarction
OR: odds ratio
PCI: percutaneous coronary intervention
SD: standard deviation
TAV: total atheroma volume
